# Assessing the Compliance of Glucose Monitoring for Patients Prescribed Antipsychotics With Intellectual Disabilities

**DOI:** 10.1192/bjo.2024.547

**Published:** 2024-08-01

**Authors:** Melissa Bremner

**Affiliations:** Woodland View Hospital, Irvine, United Kingdom

## Abstract

**Aims:**

To assess the compliance of glucose monitoring for patients prescribed antipsychotics in the local outpatient Learning Disability Team.

**Methods:**

A review was conducted of all outpatients seen in a 6 week period during September and October 2023. Each patient was reviewed to check their diagnosis or diagnoses, the antipsychotic medication they are currently on and if they have had the required tests done. These tests were considered, as per the NICE guidelines, to be a plasma glucose test or HbA1c test. It was checked to confirm if these tests had been carried out within the past 12 months, as per NICE.

**Results:**

- 46/79 patients seen in a 6 week period at the outpatient clinic were found to be currently prescribed an antipsychotic.
- 30 of those prescribed an antipsychotic were on risperidone (65%).
- Of those prescribed any antipsychotic, 18 out of 46 had not had their glucose or HbA1c checked within the past 12 months (39%).
- This therefore demonstrates 61% of patients on an antipsychotic had appropriate glucose monitoring within the time period audited.

**Conclusion:**

Monitoring glucose levels for patients on antipsychotic medication is very important for patients with an Intellectual Disability. Patients in this cohort are known to be more likely to have diabetes and obesity than the general population. In addition, there are higher levels of inactivity and multi-morbidity. It is also important to note that over-prescribing of psychotropic medication to individuals with learning disabilities, particularly antipsychotic medications such as risperidone and olanzapine, may be contributing to levels of obesity and diabetes within this population.

NICE guidelines state that for patients prescribed an antipsychotic, plasma glucose or HbA1c should be checked 3 months after commencement of treatment, and then every 12 months whilst on treatment. For olanzapine and clozapine, levels should be checked after 1 month of commencing treatment. Symptoms of hyperglycaemia should also be asked about (such as polydipsia, polyuria and increased appetite).

The results from this audit demonstrate there is definitely room for improvement in our monitoring of glucose levels for these patients. From discussing this with colleagues, it appears that a multidisciplinary approach is needed to promote this change.

Going forward, therefore, interventions should include asking the nursing staff within the outpatient team to monitor for increased appetite, polydipsia and polyuria amongst patients, especially if they are on an antipsychotic. Additionally, for any patients seen in OPC who are prescribed an antipsychotic, it should be routinely checked when they last had a glucose or HBA1c test. If this was not within the past 12 months, this should be carried out by the GP or another appropriate member of the team.

Overall, the physical health of our patients with Intellectual Disabilities is paramount. Given the nature of their Intellectual Disability and depending on the severity, it may be very challenging for them to identify any new symptoms of diabetes themselves or to report these to carers. Therefore, when prescribing antipsychotic medication, it is vital to monitor the effects of this to ensure optimal patient care utilising a multidisciplinary team approach.

